# The mediating role of school effectiveness in the relationship between transformational leadership and workplace exclusion

**DOI:** 10.3389/fpsyg.2024.1475156

**Published:** 2024-10-04

**Authors:** Ayhan Kandemir

**Affiliations:** Ministry of Education, Bolu, Türkiye

**Keywords:** school, school effectiveness, teacher, transformational leadership, Türkiye, workplace exclusion

## Abstract

The purpose of this study is to reveal the mediating role of school effectiveness in the relationship between transformational leadership and workplace exclusion. The sample of the study consisted of 410 teachers working in primary, secondary and high schools in Bolu/Türkiye province center in the 2023–2024 academic year. Correlational design was used in the study and path analysis was used to reveal the predictive relationships between variables. As a result of the study, it was seen that the hypotheses put forward in line with the model put forward were confirmed. In this context, it was concluded that transformational leadership has a significant positive effect on school effectiveness (Hypothesis 1), school effectiveness has a significant negative effect on workplace exclusion (Hypothesis 2), transformational leadership has a significant negative effect on workplace exclusion (Hypothesis 3) and finally school effectiveness has a mediating role in the effect of transformational leadership on workplace exclusion (Hypothesis 4). In the context of the results, suggestions were made such as providing in-service courses for school administrators on transformational leadership and school effectiveness, preventing exclusion at work by giving teachers the opportunity to develop themselves and participate in the decisions taken.

## Introduction

It is known that leadership styles are important for organizations to achieve the goals they set and that effective leadership plays a major role in achieving the goals of organizations. Leadership, which is one of the important concepts, can be defined as the process of mobilizing those who are behind them in line with a specific goal with these characteristics of individuals with high analytical and intuitive power, empathy, and expertise (Buil et al., 2018).

Today, with the rapid pace of change, a racing environment has emerged in the world and it has become important to realize change and change on time. Leadership (Demirtaş and Şama, 2016) and especially transformational leadership can be considered important for organizations to realize this change (Yu and Jang, 2024). Because while transformational leaders support innovation in products and technologies, they also lead changes in the strategy, mission, culture and structure of organizations (Samson et al., 2021). Transformational leadership triggers the needs that are important to those around them by making them realize the importance of their task (Yao et al., 2024). The missions of transformational leadership include inspiring and encouraging those around them and focusing on developing shared commitment and vision by bringing about positive change in community culture (Pineda, 2023). They coach their followers and empower them by supporting them in learning and personal development (Andoka et al., 2024; Harms and Crede, 2010; Kartika, 2024; Lai et al., 2020), and they positively influence their followers in terms of performance as they strategically anticipate the future and create an exciting team spirit (Joo and Lim, 2013). In addition, transformational leaders create an effective interaction between themselves and their followers by appealing to the feelings of individuals and establishing an emotional bond with them (Barutçugil, 2014). They have a vision for the future, can see changes in the organizational environment, and have the ability to motivate and inspire their members (Andoka et al., 2024). Truly transformational leaders are a directive, sensitive and forward-looking concept (Nguyen et al., 2023) that is necessary to bring about change in areas such as human resources, tools, infrastructure, finance, etc. to achieve targeted business results. Similar to these explanations, Hay (2006) explains the characteristics of transformational leaders as visionary, risk-taking, having effective communication skills, enthusiastic, open to learning, inspiring others, giving importance to cooperation, caring about the individual needs of employees, etc. When the relevant literature is examined, it is seen that transformational leadership has positive effects in organizations. For example; Alzoraiki et al. (2024) found that transformational leadership positively affects school culture and teaching performance; Jiatong et al. (2022) found that transformational leadership has a positive effect on affective organizational commitment and job performance; Yu and Jang (2024) found that transformational leadership has a positive effect on teachers’ work. When the research results are examined, it is seen that transformational leadership has a positive effect on many aspects such as organizational culture, organizational commitment and job performance. From this perspective, it can be interpreted that transformational leaders have a positive impact on the organization, creating a positive climate in the organization and reducing negativities such as organizational exclusion.

As a frequently used concept, ostracism is the exclusion, ignoring, disregarding and isolation of a person from other individuals or groups, often without giving a reason (Williams, 1997). According to another definition, ostracism is a distressing emotion for the individual, a situation that reduces the individual’s self-perception and can take away the sense of control in interaction with others (Sulea et al., 2012). Ostracism can be considered as a hidden pain that reduces the motivation of employees and affects their attitudes (Li and Zhang, 2019). On the other hand, ostracism in the workplace is a situation in which an individual or group is rejected or ignored by another individual or group to the extent that it constitutes an obstacle in terms of job success, positive relationship or reputation (Hitlan et al., 2006). In other words, exclusion in the workplace causes employees to be ignored in the organization they work for and, consequently, to become depressed and unable to meet their basic needs (Xing and Li, 2022). It is understood that individuals who are exposed to exclusion in the workplace; group loyalty, job satisfaction and organizational citizenship perceptions decrease (Mohsin et al., 2022; Qian et al., 2019; Zhang et al., 2023), cause problems such as depression, anxiety, and emotional exhaustion (Wang G.H. et al., 2023; Yang and Wei, 2018) and are one of the main causes of stress in organizations (Liu et al., 2013; Noor and Abbas, 2024). This situation is also reflected in the studies conducted. For example, Wang L. M. et al. (2023) concluded in their study that increased information sharing in the workplace decreases exclusion and burnout. Noor and Abbas (2024) found that ostracization in the workplace depletes resources such as self-esteem and self-control. At this stage, it can be interpreted that exclusion can cause negative consequences for organizations and prevent positive situations such as school effectiveness.

Effectiveness is defined as “*the degree to which the organization achieves its goals*” (Barnard, 1938, cited in Çiftçi, 2023) or the state of achieving success in the results obtained by using effective methods in terms of revealing the determined goals, reaching the necessary resources and adapting to the environment (Ada and Baysal, 2012). From this point, it is seen that effectiveness is an important situation for organizations. Because it is known that organizations maintain their existence when they are effective and sufficient (Aydın, 2010). It is understood that this situation is also valid for schools, which are important organizations. Effective schools are characterized by clear educational goals, systematic evaluation, safe school climate, quality, and holistic development of students (Edmonds, 1979, as cited in Diş, 2023). In other words, effective schools are schools where students are supported with all areas of development at the highest rate (Abdurrezzak, 2015). In effective schools, factors such as teacher motivation (Khun-Inkeeree et al., 2022), strong leadership, positive school culture, meeting students’ needs, promoting social justice, and parent involvement are prominent (Javornik and Klemenčič Mirazchiyski, 2023). It is seen that there are different stakeholders in the formation of these environments in effective schools. Zigarelli (1996) stated that factors such as teacher quality, leadership and communication skills of the administrator, teacher participation, strong school culture, and participation of families are important in effective schools. Weindling (1989; as cited in Al-Mekhlafi and Osman, 2022) similarly listed the characteristics of effective schools as emphasizing learning, shared vision and expectations, parent-school collaboration, professional leadership, staff development, positive reinforcement, etc. As can be seen, many factors play a role in effective schools. This situation has also manifested itself in research. For example, Kale (2023) concluded in his study that there is a positive significant relationship between school effectiveness and trust in instructors; Özgenel (2020) concluded in his study that school climate predicts school effectiveness. Again, Özgenel and Koç (2020) found that there is a significant relationship between teachers’ professional commitment and school effectiveness.

Since transformational leaders strategically envision the future and create excitement in the team spirit, it is seen that they positively increase the performance of their followers (Joo and Lim, 2013) and adopt management based on learning and development (Loyless, 2023). It can be said that this situation also manifests itself in schools. Transformational leaders enable teachers to develop up-to-date teaching and learning approaches, allowing them to develop individually and, accordingly, to educate students according to the requirements of the age (Celep, 2004). Transformational leadership aids school and community development through a culture that fosters innovative capacity and moral commitment to student achievement, sustained performance and school community development (Alzoraiki et al., 2024). At this point, it can be interpreted that the transformational leadership characteristics of administrators have a positive effect on schools to be effective schools. Because it is seen that schools that try to increase their academic levels make efforts to increase their leadership capacities, and school principals who serve as administrators make efforts to transform the school culture (Marks and Printy, 2003). In addition, transformational leaders make individual contributions to their colleagues by sharing risks and responsibilities with them (Moolenaar et al., 2010). In addition, transformational leaders contribute to the development of employees by supporting them, and they produce appropriate solutions to problems by expressing their wishes (Bass, 1996). Thus, it is interpreted that negative attitudes such as emotional exhaustion, high stress, and exclusion in the workplace (Lane, 2017), which lead to problems such as achieving goals, etc., will decrease in organizations where transformational leadership characteristics are seen. When the related literature is examined, it is seen that there is no study investigating the effect of transformational leadership on workplace exclusion and the mediating role of school effectiveness. This situation makes the research important. In addition, when the effect of transformational leadership on workplace exclusion is revealed, it will be easier to encourage school administrators to become transformational leaders. In addition, investigating the mediating role of school effectiveness in the effect of transformational leadership on workplace exclusion makes the research important in terms of revealing the characteristics of effective schools. Finally, it is thought that the research is important in terms of shedding light on both decision makers and future studies.

## Method

### Study group

The study group of the current study consisted of 410 volunteer teachers working in primary, secondary and high schools in the center of Bolu/Türkiye in the second semester of the 2023–2024 academic year. The type of sampling in which the researcher saves time, money and effort by easily accessing the group of the size needed by the researcher is accepted as convenient sampling (Büyüköztürk, 2010). Furthermore, the selection of schools was based on their different levels, types (vocational, science and Anatolian high schools, middle schools, imam-hatip middle schools, etc.) and social environments. Thus, it was aimed to reach different opinions by reaching teachers working at different levels of education. Of the teachers who participated in the study; 262 (63.7%) were female, 149 (36.3%) were male; 310 (75.6%) had undergraduate education, 100 (24.4%) had postgraduate education; 68 (16.6%) had 0–10 years, 192 (46.8%) 11–20 years; 119 (29.0%) had 21–30 years of seniority and 31 (7.6%) had 31 or more years of seniority; 116 (28.3%) worked in primary schools, 133 (32.4%) in secondary schools and 161 (39.3%) in high schools; 387 (94.4%) were married and 23 (5.6%) were single.

### Research model and hypotheses

In the study, correlational design (Dancey and Reidy, 2017) was used to determine the relationship between transformational leadership, school effectiveness and workplace exclusion according to teacher perceptions, and path analysis was used to reveal the predictive relationships between variables.

**Figure 1:**
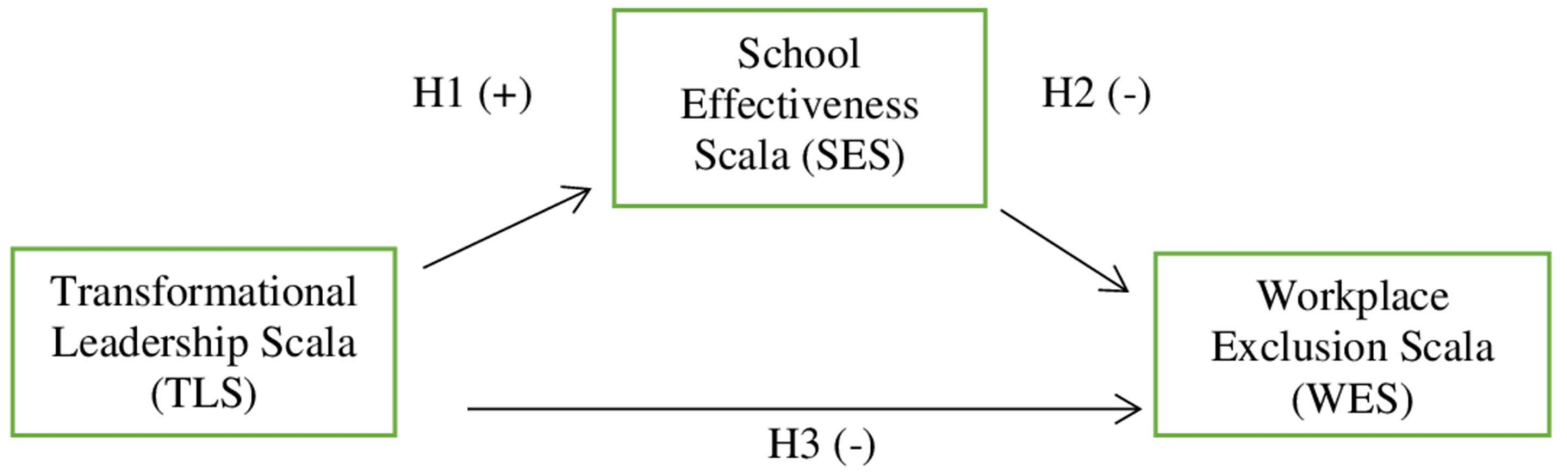
Research model.

The model created for transformational leadership, workplace exclusion and school effectiveness in the context of the current study is given in Figure 1.

When Figure 1 is examined, the research model was determined as transformational leadership (independent variable), school effectiveness (mediating variable), and workplace exclusion (dependent variable). In line with the determined model, the following hypotheses were included;

*Hypothesis 1*: Transformational leadership has a significant positive effect on school effectiveness.

*Hypothesis 2*: School effectiveness has a significant negative effect on workplace exclusion.

*Hypothesis 3*: Transformational leadership has a significant negative effect on workplace exclusion.

*Hypothesis 4*: School effectiveness has a mediating role in the effect of transformational leadership on workplace exclusion.

### Data collection

The research data were collected face-to-face from volunteer teachers with the permission of Bolu Abant İzzet Baysal University Human Research Ethics Committee in Social Sciences with protocol number 2024/69. During the data collection phase, school administrations were first informed. Then, participating teachers were briefly informed about the study by the researcher and data were collected from volunteer teachers. The data obtained were transferred to digital media and made ready for analysis.

### Data analysis

It is important that the sample size is sufficient to determine any effect between variables in research. In order to reveal indirect effects between variables, 115–285 participants are recommended for mediation analysis (Fritz and MacKinnon, 2007). In the current study, a sample size of 410 participants, which is larger than this range, was included. JAMOVI analysis program was used in the analysis of the data. Before the analysis, Skewness and Kurtosis distributions were checked for normality distribution. In the distributions, the data belonging to 4 participants with extreme values (those with a scale mean score of 4 and above) in the WES in the exclusion at work scale were excluded from the data set, and no extreme data were found in other scales. Since the scales met the normality condition, no rotation was performed on the data. The analysis continued with the data of 410 participants. When the results were examined, it was found that the scales of TLS (Skewness = −,913, sd = ,121; Kurtosis = ,573, sd = ,240), WES (Skewness = −1,416, sd = ,121; Kurtosis = 1,964; sd = ,240), SES (Skewness = −1,107, sd = ,121; Kurtosis = 1,440; sd = ,240), the skewness and kurtosis values of the scales were between −2 and +2 and therefore it was accepted that the distributions met the normality condition (George and Mallery, 2003; Şencan, 2005). The results were evaluated at 95% confidence interval and *p* < 0.05 was considered significant. In addition, the reliability of the scales was evaluated according to Chronbach’s alpha coefficient (*α* ≥ 0.70; Eymen, 2007; Şencan, 2005).

### Data collection tools

Three different scales were used in the study. Information about the scales is as follows:

The short transformational leadership scale was developed by Berger et al. (2012), and adapted into Turkish by Okan and Okan (2021). The scale consisted of 8 items and one dimension, and the scale was prepared in a five-point Likert-type scale including “Strongly disagree” (1), “Disagree” (2), “Undecided” (3), “Agree” (4) and “Strongly disagree” (5). There are no reverse items in the scale. First, the linguistic equivalence of the scale was ensured. Then, the researchers conducted validity (*χ*^2^ = 2.51; *p* < 0.001; RMSEA = 0.06; RMR = 0.009; NFI = 0.98; CFI = 0.992; GFI = 0.969) and reliability (Cronbach’s Alpha: ,97) studies and concluded that the scale was valid and reliable. In the current study, Cronbach’s Alpha value was again found to be .97.

The perceived school effectiveness scale was developed by Hoy (2014) and adapted into Turkish by Yıldırım and Ada (2018). The scale consisted of 8 items and a single dimension, and the scale was prepared in a six-point Likert scale including “Strongly Disagree” (1), “Disagree” (2), “Undecided” (3), “Somewhat Agree” (4), “Agree” (5) and “Strongly Disagree” (6). There are no reverse items in the scale. The researchers conducted validity (*χ*^2^ = 3.06; *p* < 0.001; RMSEA = 0.063; RMR = 0.045; NFI = 0.98; CFI = 0.99; GFI = 0.97), language equivalence coefficient (0.708) and reliability (Cronbach’s Alpha:,86) studies and concluded that the scale was valid and reliable. In the current study, the Cronbach’s Alpha value of the scale was found to be.92.

The workplace exclusion scale was developed by Ferris et al. (2008) and adapted into Turkish by Kurum and Erdemli (2022). The scale consisted of 10 items and a single dimension, and the scale was prepared in a seven-point Likert-type scale including “Strongly Disagree” (1), “Disagree” (2), “Somewhat Disagree” (3), “Neither Agree nor Disagree” (4), “Somewhat Agree” (5), “Agree” (6) and “Strongly Agree” (7). There are no reverse items in the scale. First, the linguistic equivalence of the scale was ensured. Then, the researchers conducted validity (*χ*^2^ = 2.03; *p* < 0.001; RMSEA = 0.06; NNFI = 0.98; NFI = 0.98; AGFI = 0.92; GFI = 0.95) and reliability (Cronbach’s Alpha: ,88) studies and concluded that the scale was valid and reliable. In the current study, the Cronbach’s Alpha value of the scale was found to be 0.93.

When the Cronbach’s Alpha values of the scales were analyzed, it was concluded that the reliability levels of the three scales were sufficient (Eymen, 2007; Şencan, 2005).

### Findings

The relationship between transformational leadership scale (TLS), school effectiveness scale (SES) and workplace exclusion scale (WES) was analyzed by Pearson Correlation analysis and given in Table 1.

**Table 1:** The relationship between the scales.

	Descriptive statistics					Correlations		
Variable	*N*	Min.	Max.	x̄	SD	1	2	3
1. TLS	410	1.00	5.00	3.78	0.93	1		
2. WES	410	1.00	3.80	1.40	0.52	−0.153^*^	1	
3. SES	410	1.00	6.00	4.55	0.92	0.474^**^	−0.289^**^	1

^*^Correlation significant at 0.05 level. ^*^Correlation significant at 0.01 level. *n*, sample number, X̄, mean, SD, standard deviation.

Table 1 shows that teachers’ perceptions of transformational leadership (x̄ = 3.78; SD = 0.93) and school effectiveness (x̄ = 4.55; SD = 0.92) are higher than their perceptions of exclusion at workplace (x̄ = 1.40; SD = 0.52). Correlation coefficient values are also given in Table 1. Correlation coefficient values; 0.10–0.29 is interpreted as small, 0.30–49 as medium, and 0.50–1.00 as large effect (Cohen, 1988; Cohen, 1992). Accordingly, when Table 1 is analyzed, it is concluded that there is a negative and low level relationship between transformational leadership and workplace exclusion (r = −0.153; *p* < 0.01), a positive and medium level relationship between transformational leadership and school effectiveness (r = 0.474; *p* < 0.01), and a negative and low level relationship between school effectiveness and workplace exclusion (r = −0.289; *p* < 0.01).

### Reliability analysis

The reliability of the scales in the model was evaluated with Cronbach’s Alpha coefficient, McDonald’s Omega (*ω*) and Composite Reliability (CR) internal consistency coefficients. The internal consistency reliability of the scales in the model was evaluated with Cronbach’s Alpha Coefficient and Cronbach’s alpha values greater than 0.70 at 95% confidence interval for each of the constructs were taken as a reference (Eymen, 2007; Şencan, 2005). It is stated that the internal consistency value should be 0.70 and above to ensure the reliability of a scale. As a result of the analysis, the internal consistency coefficient of the Transformational Leadership Scale was 0.97, McDonald’s Omega (ω) = 0.97, CR (Composite Reliability) = 0.70, the internal consistency coefficient of the Workplace Exclusion Scale was 0.93, McDonald’s Omega (ω) = 0.94; CR (Composite Reliability) = 0.94; and the internal consistency coefficient of the School Effectiveness Scale was 0.92, McDonald’s Omega (ω) = 0.94, CR (Composite Reliability) = 0.94 (Table 2). When the Cronbach’s Alpha values of the scales were analyzed, it was concluded that the reliability levels of the three scales were sufficient (Büyüköztürk, 2010; DeVellis, 2017; Eymen, 2007; Kline, 2015; Şencan, 2005).

**Table 2:** Validity and reliability of the scales.

Scale	Item	Factor Loading	Cronbach’s Alpha	ω	AVE	CR
Transformational leadership scala (TLS)	TLS 1	0.89				
	TLS 2	0.91				
	TLS 3	0.90				
	TLS 4	0.89				
	TLS 5	0.90	0,97	0.97	0,88	0.70
	TLS 6	0.93				
	TLS 7	0.92				
	TLS 8	0.91				
School effectiveness scala (SES)	SES 1	0.75				
	SES 2	0.78				
	SES 3	0.84				
	SES 4	0.88	0,92	0.94	0,72	0.94
	SES 5	0.85				
	SES 6	0.85				
	SES 7	0.84				
	SES 8	0.82				
Workplace exclusion scala (WES)	WES 1	0.64				
	WES 2	0.71				
	WES 3	0.70				
	WES 4	0.80				
	WES 5	0.88	0,93	0.94	0.64	0.94
	WES 6	0.81				
	WES 7	0.89				
	WES 8	0.87				
	WES 9	0.89				
	WES 10	0.76				

### Validity analysis

It is important to provide the required sample size for structural equation modeling. Sample size affects the correct estimation of the model and the detection of specification error (Teo et al., 2013). The KMO test is the test that measures the correlations between variables and the suitability of factor analysis, and the value of the KMO test should be between 0 and 1 (Yaşlıoğlu, 2017). For this purpose, KMO sampling criterion was used for the validity analysis of the model and the KMO test result of.94 showed that the sample size was very suitable for the analysis. In addition, Bartlett’s Test of Sphericity was found as 11,602,302, *p* < 0.05.

Then, factor loadings and average variance explained (AVE) values of the variables were evaluated for convergent validity. Since there were no items with factor loadings below 0.40, no item was removed from the analysis. It was determined that the item factor loadings of the TLS scale ranged between 0.89 and 0.93, the item factor loadings of the SES scale ranged between 0.75 and 0.88, and the WES scale ranged between 0.64 and 0.89 (Table 2). AVE values are expected to be above 0.50. However, if the factor loadings of the items were less than 0.70, the AVE value was required to be 0.50 (Hair et al., 2017). Although the item factor loading was lower than 0.70 (0.64) in item 1 of the Workplace Exclusion Scale, the AVE value was found to be 0.80 (Table 2). In this case, it was seen that all scales met the convergent validity.

### Structural model evaluation

The concept of model goodness shows whether the constructed structural model reflects the data situation positively. Poor model fit reduces the reliability of the results (Kang and Ahn, 2021). In the model fit evaluation of structural equation modeling; *χ*^2^/df, NFI, TLI, CFI, SRMR and RMSEA fit indices were included. Since the chi-square statistic is easily affected by sample size, the *χ*^2^/df ratio, which is less affected by sample size, can be used (Yazıcıoğlu and Erdoğan, 2014). Therefore, first the *χ*^2^/df value of the structural equation model was found to be 1.86. Since this result is less than 2, it can be said that the model provides a good fit (Tabachnick and Fidell, 2007). Then, other fit values of the model were calculated. Table 3 shows the NFI, TLI, CFI, SRMR, and RMSEA values of the model and the model was accepted because the results obtained were between good and acceptable fit values.

**Table 3:** Model goodness-of-fit results.

Goodness of fit indices	Good fit value	Acceptable fit value	Structural model
*p* value	0.05 ≤ *p* ≤ 1.00	0.01 ≤ *p* ≤ 0.05	*p* < 0,01
*χ* ^2^ */df (sd)*	0 ≤ *χ*^2^/df ≤ 2	2 ≤ *χ*^2^*/df* ≤ 3	1,86
NFI	0.95 ≤ NFI ≤ 1.00	0.90 ≤ NFI ≤ 0.95	0.99
TLI	0.95 ≤ TLI ≤1.00	0.90 ≤ TLI ≤ 0.95	0.99
CFI	0.95 ≤ CFI ≤ 1.00	0.90 ≤ CFI ≤ 0.95	0.99
SRMR	0 < SRMR ≤0.05	0.05 < SRMR≤0.10	0.04
RMSEA	0 ≤ RMSEA≤0.05	0.05 ≤ RMSEA≤0.08	0.04

*RMSEA, root mean square error of approximation, SRMR, standardized root mean squared residual; CFI, comparative fit index; TLI, Tucker-lewis index. Cited in Erkorkmaz et al., 2013; İlhan and Çetin, 2014.

When Table 3 is examined, it is seen that the model fit is good because the SRMR value is below.80 (0.04) in the model goodness of fit indices (Teo et al., 2013). In addition, an NFI value above.80 indicates the acceptability of the model and an NFI value of.99 indicates the validity of the model (Table 3).

**Figure 2:**
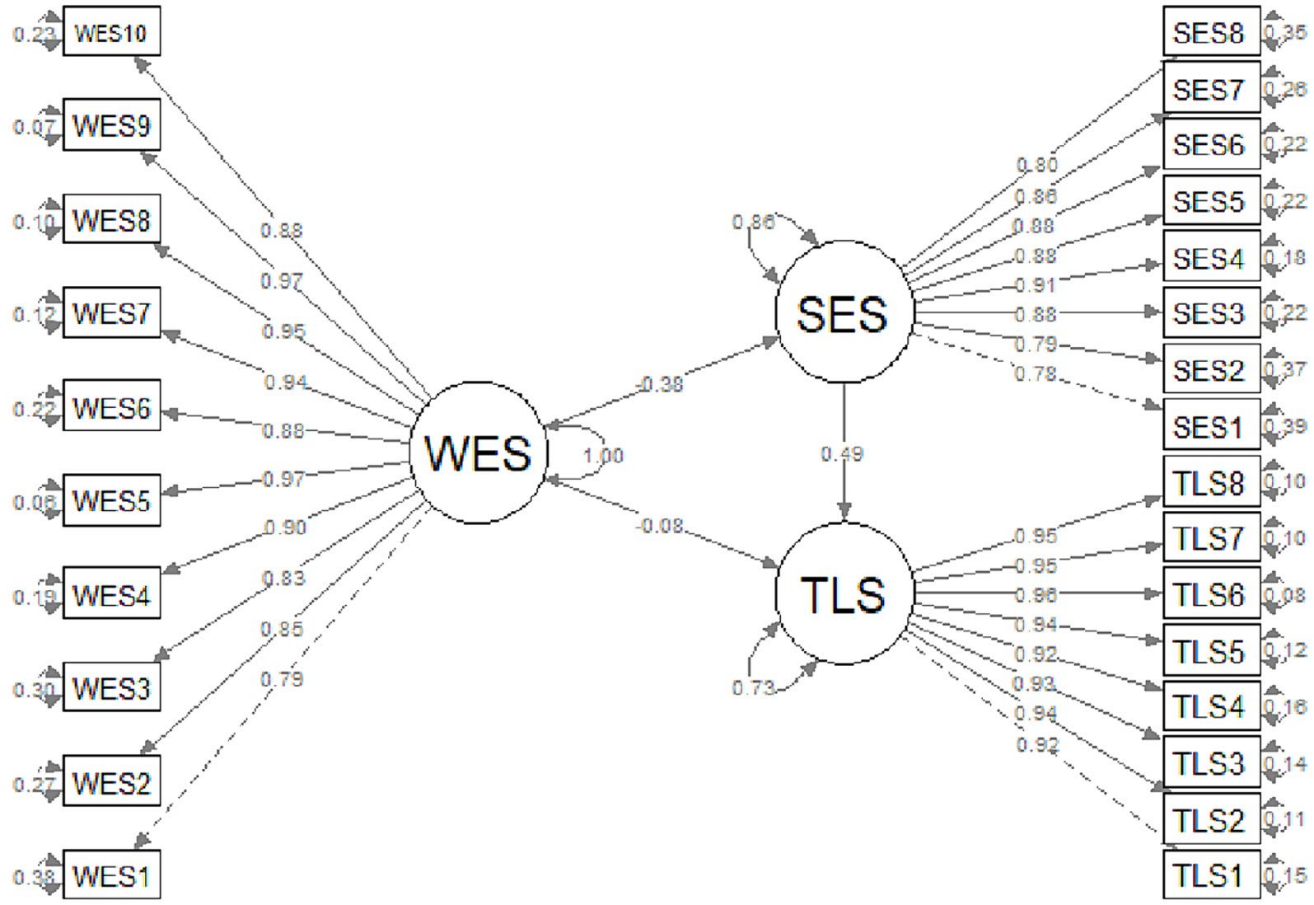
Model based on analysis results.

As a result of the analysis, it was seen that the models established in the planning stage of the research were valid *p* < 0.01. It can be said that school effectiveness is a mediating variable in the effect of transformational leadership on workplace exclusion. Because while the relationship between transformational leadership and workplace exclusion was *β* = −0.08 at the beginning, this relationship became β = −0.18 when school effectiveness was added (Table 4). This situation is also shown in Figure 2.

**Table 4:** Research model structural equation analysis results.

Relationship	β	R^2^	*p*-value	Hypothesis	Result
TLS → SES	0,47	0,22	<0.001	H1	Verified
SES → WES	−0,28	0,08	<0.001	H2	Verified
TLS → WES	−0,15	0,02	<0.001	H3	Verified
WES → SES → TLS	−0,18	0,08	<0.001	H4	Verified

## Conclusion, discussion, and recommendations

This section of the present study presents the results, discussion and recommendations in the context of the results.

In Hypothesis 1, it was assumed that transformational leadership has a positive and significant effect on school effectiveness and when the results were analyzed, Hypothesis 1 was confirmed (β = 0.47; R^2^ = 0.22; *p* < 0.001). Ermeydan and Can (2020) found that transformational leadership is a significant predictor of school effectiveness, and Sesli (2023) found that transformational leadership characteristics applied in schools positively affect school effectiveness in his study. When the related literature is examined, it is seen that there are different studies that are similar to the research results (Abdurrezzak, 2015; Akçay Güngör, 2018; Dufour et al., 2004; Tuncel, 2013). In order for schools to be effective schools and to bring about the intended changes, school leaders should have sufficient knowledge of transformational leadership principles (Dufour et al., 2004; Marks and Nance, 2007). Because school leadership is seen as one of the characteristics of effective schools (Javornik and Klemenčič Mirazchiyski, 2023; Sammons, 2010; Weindling, 1989; cited in, Al-Mekhlafi and Osman, 2022). It is seen that it will be easy for schools to reach their mission and vision if school administrators have transformational leadership characteristics. In this way, it can be thought that the administrator will have the opportunity to transform the school in line with the goals by meeting the needs of teachers, parents and students, thus increasing school effectiveness. Therefore, it can be concluded that the positive and significant effect of transformational leadership on school effectiveness is a natural result.

In Hypothesis 2, it was hypothesized that school effectiveness has a significant negative effect on workplace exclusion and the hypothesis was confirmed as a result of the study (*β* = −0.28; R^2^ = 0.08; *p* < 0.001). An effective school is a school that increases the learning outcomes and quality of students, responds to the educational needs of the society (Yumuşak and Korkmaz, 2024), and adds value to the results of students when compared to similar schools (Mortimore, 1991; as cited in Lafcı-Tor and Yavuz, 2020). The concept of school effectiveness is affected differently by many variables such as school, administrators, teachers and students (Yumuşak and Korkmaz, 2024). Mortimore (1988; as cited in Al-Mekhlafi and Osman, 2022) also states that the main determinants of school effectiveness are the leadership of school administrators and the participation of teachers in the decision-making process. Therefore, it can be said that teachers, who are seen to have an impact on school effectiveness, should have positive motivation towards the school they work for. Because teachers’ motivation has an important role on teacher performance and thus on students (Gemeda and Tynjälä, 2015). It is seen that teachers’ having positive motivation towards school will increase their success and thus school effectiveness. Thus, in effective schools, it can be thought that exclusion, which causes negative situations such as depression, thoughts of uselessness, anxiety, violence (Yang, 2012), inability to express their views (Luo et al., 2023), will decrease. Because it can be said that having employees who have high job satisfaction and organizational citizenship and who can express their opinions openly will make it easier for the school to be an effective school. Therefore, in the current study, it can be thought that school effectiveness has a negative effect on exclusion at work.

In Hypothesis 3, it was hypothesized that transformational leadership has a significant negative effect on workplace exclusion and the hypothesis was confirmed in the context of the results (*β* = −0.15; R^2^ = 0.02; *p* < 0.001). Robbins and Judge (2012) state that transformational leaders have a significant impact on their followers by integrating the goals of the followers and the goals of the organization, and that they care about and support the individual development of their subordinates (Robbins and Judge, 2012). Yukl (2018) states that transformational leaders are leaders who develop the skills and confidence of their followers. Subordinates who have such leaders in organizations increase their organizational commitment and satisfaction levels (McShane and Von Glinow, 2016) and their organizational citizenship is positively affected (Li et al., 2023). Because transformational leaders have a vision for the future and can see the changes in the environment. They motivate and inspire their members and help them achieve higher goals (Andoka et al., 2024). It can be said that this situation will reduce the perception of exclusion at work, which is defined as a form of exploitation (Lance Ferris et al., 2019; Wu et al., 2019) that causes negative situations such as a decrease in job satisfaction, an increase in thoughts of leaving the job, and deterioration in sleep quality. Therefore, it can be said that the significant negative effect of transformational leadership on workplace exclusion in the current study is a normal situation.

The mediating role of school effectiveness in the effect of transformational leadership on workplace exclusion constituted the 4th hypothesis of the study, and the hypothesis was confirmed in the context of the results obtained (β = −0.18; R^2^ = 0.08; *p* < 0.001). Transformational leaders enable the psychological, managerial, and work-oriented development of their environment, thus enabling their personal development. In addition, in this process, they provide a sustainable feedback mechanism that is important in the development of their environment (Jung et al., 2009; as cited in Yılmaz and Kasımoğlu, 2024). They encourage and inspire people to achieve goals (Florek-Paszkowska and Hoyos-Vallejo, 2023). It is seen that this situation plays an important role in the emergence of effective schools (Çevik, 2023) where the views of stakeholders are valued, ideas are clearly put forward, and the school culture supports personal and social development. In this case, it is seen that the transformational leadership characteristics of school administrators have a positive effect on schools to be effective schools by influencing the teachers around them. Javornik and Klemenčič Mirazchiyski (2023) also stated that factors such as strong leadership, positive school climate, etc. contribute to effective schools. It can be thought that this situation will reduce teachers’ perceptions of exclusion in the workplace (Hitlan et al., 2006; Mohsin et al., 2022; Qian et al., 2019; Zhang et al., 2023), which negatively affects their perceptions of job success, positive relationship, reputation, group commitment, job satisfaction and organizational citizenship. Thus, it can be interpreted that transformational leadership plays a mediating role of school effectiveness on workplace exclusion.

As a result of the study, it can be said that the fact that transformational leadership is effective on workplace exclusion and school effectiveness makes the study important. The fact that leadership is among the characteristics of effective schools (Javornik and Klemenčič Mirazchiyski, 2023) can bring transformational leadership to the forefront in policies and practices in Türkiye. It can be said that with the development of policies for transformational leadership, negative situations in schools will be eliminated and it will be easier for schools to become effective schools. This situation May lead decision makers in Türkiye to develop policies to equip school administrators with the characteristics of being transformational leaders. In fact, in the “2023 Vision” (2023 Eğitim Vizyonu, 2018), it is stated that postgraduate education will be given importance for the professional development of administrators and teachers. It can be said that by directing administrators to graduate education, administrators will have more knowledge about leadership and their awareness of transformational leadership will increase. In the same document, the fact that the goals for the development of teachers and students along with administrators are also stated can be interpreted as giving importance to school effectiveness. The result that both transformational leadership and school effectiveness reduce exclusion in the workplace can also guide future education policies. It can be interpreted that these issues will be given more importance in future education policies.

In the context of these results, suggestions can be made for practitioners to provide in-service courses for school administrators on transformational leadership and school effectiveness, to prevent exclusion in the workplace by giving teachers the opportunity to develop themselves and participate in the decisions taken. For practitioners, suggestions can be made to conduct similar studies in preschool schools or in different provinces.

## Limitations

Although the study has reached important conclusions, it also has some limitations. First of all, only Bolu province center was taken as the study population. This does not represent the whole of Türkiye. Therefore, future studies can be expanded to include different provinces. Secondly, only transformational leadership was included among leadership types in the study. Considering that there are many different types of leadership, studies can be conducted on different leadership styles. Finally, this study is related to transformational leadership, school effectiveness and workplace exclusion. Considering that many factors are important on schools, studies on different subjects can be included.

## Data Availability

The raw data supporting the results of the article can be made available if there is a request.
