# Corrigendum: The mediating role of school effectiveness in the relationship between transformational leadership and workplace exclusion

**DOI:** 10.3389/fpsyg.2024.1519426

**Published:** 2025-01-20

**Authors:** Ayhan Kandemir

**Affiliations:** Ministry of Education, Bolu, Türkiye

**Keywords:** school, school effectiveness, teacher, transformational leadership, Türkiye, workplace exclusion

In the published article, there were several errors. In the sections *Method*, and *Conclusion, discussion, and recommendations*, a comma (,) was mistakenly used in several places instead of a period (.) in some places to indicate numerical data.

Additionally, in section *Method, Findings, Paragraph 2*, the r number stated as “474” was incorrect. The corrected sentence should be written as shown below.

“Accordingly, when Table 1 is analyzed, it is concluded that there is a negative and low level relationship between transformational leadership and workplace exclusion (r = −0.153; *p* < 0.01), a positive and medium level relationship between transformational leadership and school effectiveness (r = 0.474; *p* < 0.01), and a negative and low level relationship between school effectiveness and workplace exclusion (r = −0.289; *p* < 0.01).”

Additionally, in section *Method, Validity Analysis*, and *Paragraph 2*, some values were written incorrectly. Instead of “Then, factor loadings and average variance explained (AVE) values of the variables were evaluated for convergent validity. Since there were no items with factor loadings below 0.40, no item was removed from the analysis. It was determined that the item factor loadings of the TLS scale ranged between 0.89 and 0.93, the item factor loadings of the SES scale ranged between 0.75 and 0.88, and the WES scale ranged between 0.69 and 0.089 (Table 2). AVE values are expected to be above 0.50. However, if the factor loadings of the items were less than 0.70, the AVE value was required to be 0.50 (Hair et al., 2017). Although the item factor loading was lower than 0.70 (0.65) in item 1 of the Workplace Exclusion Scale, the AVE value was found to be 0.80 (Table 2). In this case, it was seen that all scales met the convergent validity.”

The corrected text is shown below:

“Then, factor loadings and average variance explained (AVE) values of the variables were evaluated for convergent validity. Since there were no items with factor loadings below 0.40, no item was removed from the analysis. It was determined that the item factor loadings of the TLS scale ranged between 0.89 and 0.93, the item factor loadings of the SES scale ranged between 0.75 and 0.88, and the WES scale ranged between 0.64 and 0.89 (Table 2). AVE values are expected to be above 0.50. However, if the factor loadings of the items were less than 0.70, the AVE value was required to be 0.50 (Hair et al., 2017). Although the item factor loading was lower than 0.70 (0.64) in item 1 of the Workplace Exclusion Scale, the AVE value was found to be 0.80 (Table 2). In this case, it was seen that all scales met the convergent validity.”

Lastly, the **Author Contributions** were not complete in the published article. Instead of “AK: Writing – original draft.”

This section should be written as shown below:

